# Effects of MRP8, LPS, and Lenalidomide on the Expressions of TNF-****α****, Brain-Enriched, and Inflammation-Related MicroRNAs in the Primary Astrocyte Culture

**DOI:** 10.1155/2013/208309

**Published:** 2013-09-21

**Authors:** Ahmed Omran, Muhammad Usman Ashhab, Na Gan, Huimin Kong, Jing Peng, Fei Yin

**Affiliations:** ^1^Department of Pediatrics, Xiangya Hospital of Central South University, Changsha, Hunan 410008, China; ^2^Departments of Pediatrics and Neonatology, Suez Canal University, Ismailia 41522, Egypt

## Abstract

Astrocytes are now recognized as a heterogeneous class of cells with many important and diverse functions in healthy and diseased central nervous system (CNS). MicroRNAs (miRNAs) are small, noncoding RNAs which may have key roles in astrocytes activation in response to various stimuli. We performed quantitative real-time PCR (qPCR) to detect changes in the expressions of brain-enriched miRNAs (124, 134, 9, 132, and 138), inflammation-related miRNAs (146a, 21, 181a, 221, and 222), and tumor necrosis factor alpha (TNF-**α**) in the rat primary astrocyte cultures after stimulation with myeloid-related protein 8 (MRP8) and lipopolysaccharides (LPS). Further, we inhibited the expression of TNF-**α** in the astrocytes by using TNF-**α** inhibitor (lenalidomide) and tested for the first time the effect of this inhibition on the expressions of the same tested miRNAs. Stimulation of the astrocytes with MRP8 or LPS leads to significant upregulation of miRNAs (124, 134, 9, 132, 146a, 21, 181a, 221, and 222), while miRNA-138 was downregulated. TNF-**α** inhibition with lenalidomide leads to opposite expressions of the tested miRNAs. These miRNAs may play an important role in activation of the astrocytes and may be a novel target for cell-specific therapeutic interventions in multiple CNS diseases.

## 1. Introduction

Astrocytes are dynamic cells considered as the most abundant cells in the central nervous system (CNS). In the last 3 decades it has become clear that astrocytes are responsible for a wide variety of essential and complex functions in the healthy CNS [1]. Astrocyte dysfunction is becoming a recognized feature in multiple brain diseases including migraine, epilepsy, leukodystrophies, inflammatory demyelinating diseases, infections, brain edema and metabolic disorders, metal intoxications, neurodegenerative disorders, and schizophrenia [2, 3].

In response to inflammatory or other pathological conditions, astrocytes leave their quiescent state and become activated in a process known as astrogliosis [4]. This is associated with initiation and regulation of CNS immune response via the release of proinflammatory cytokines tumor necrosis factor alpha (TNF-*α*), interleukin 1-*β* (IL-1*β*), and interleukin-6 (IL-6) [5].

MicroRNAs (miRNAs) are small, nonprotein-coding RNA molecules that modulate gene translation. Their expressions are altered in multiple CNS, cardiovascular, and chronic diseases [6–8]. Sixty to seventy percent of the known miRNAs are expressed in the brain, while some of those are enriched or unique to neuronal tissues [9, 10]. MiRNAs are also recognized as vital regulators in immunity and inflammation making some of the identified miRNAs be inflammation-related [11, 12]. Expressional levels of miRNAs changed when the astrocytes are subjected to different stimuli such as lipopolysaccharide (LPS) and interferon gamma (IFN-*γ*) [13] or oxygen-glucose deprivation (OGD) [14].

Activated astrocyte is a major source of TNF-*α* in the inflamed CNS, which plays an important role in the pathogenesis of multiple CNS diseases such as brain trauma, ischemic injury, multiple sclerosis, and Alzheimer's disease [15].

Lenalidomide is a derivative of thalidomide that belongs to a class of drugs known as immunomodulating drugs [16]. With respect to the parent drug, thalidomide, lenalidomide is a more potent inhibitor of TNF-*α* and has a weaker antiangiogenic effect. 

In this study, we demonstrate the changes in the expressional levels of brain-enriched miRNAs (124, 134, 9, 132, and 138) and inflammation-related miRNAs (146a, 21, 181a, 221, and 222), when the astrocytes are subjected to stimulation with proinflammatory mediators (myeloid-related protein 8 (MRP8) and LPS). Further, we examined for the first time the effect of lenalidomide (TNF-*α* inhibitor) followed by stimulation with MRP8 and LPS on the expressions of the same miRNAs.

## 2. Materials and Methods

### 2.1. Primary Astrocyte Culture

Astrocytes were obtained from cerebral cortices of male neonatal Sprague-Dawley (SD) rats. The animals were decapitated and the brains were fetched immediately and placed in phosphate-buffered saline (PBS) at 4°C. After removing the meninges and blood vessels, the tissues were minced, washed, centrifuged, and incubated in 0.025% (w/v) trypsin and 60 mg/mL DNase for 15–20 minutes. High-glucose Dulbecco's Modified Eagle Medium (DMEM) (Highclone, USA) containing 10% (v/v) fetal bovine serum (FBS) is added to complete the trypsinization. Cells were cultured in DMEM supplemented with 10% FBS, 2 mM L-glutamine, 100 units/mL penicillin, and 100 *μ*g/mL streptomycin and were maintained at 37°C under >90% humidity and 5% CO_2_. The culture medium was renewed twice a week till cells reached confluence then the cells were purified by repeated trypsinization and inoculated at a density of 2.5 × 10^4^ cells/cm^2^ at least 4 times. More than 95% of the cultured cells were astrocytes as identified by immunofluorescent staining for glial fibrillary acidic protein (GFAP). Experiments were carried out 21 days after culturing of the astrocytes.

### 2.2. Stimulation of the Astrocytes with MRP8 and LPS

Part of the prepared astrocytes was divided into three groups.

*Group A*. Astrocytes are cultured without serum DMEM medium for 30 hours to detect the expressions of the tested brain-enriched miRNAs, inflammation-related miRNA, and TNF-*α* in the resting astrocytes.

*Group B*. Astrocytes are cultured without serum DMEM medium for 6 hours then with DMEM and MRP8 (0.5 *μ*g/mL) for 24 hours [17], to detect the effect of astrocytes stimulation with MRP8 on the expressions of the tested brain-enriched miRNAs, inflammation-related miRNAs, and TNF-*α*.

*Group C*. Astrocytes are cultured without serum DMEM medium for 6 hours then with DMEM and LPS ((1000 ng/mL), SIGMA, USA) for 24 hours [18], to detect the effect of astrocytes stimulation with LPS on the expressions of the tested brain-enriched miRNAs, inflammation-related miRNAs, and TNF-*α*. 

### 2.3. Stimulation of the Astrocytes with MRP8 and LPS after TNF-*α* Inhibition with Lenalidomide

Other part of the prepared astrocytes was divided into two other groups.

*Group D*. Astrocytes are cultured without serum DMEM medium containing TNF-*α* inhibitor lenalidomide ((2 *μ*M), Selleckchem, USA) for 6 hours and then cultured with DMEM medium with MRP8 for 24 hours to detect the effect of TNF-*α* inhibition on the tested brain-enriched and inflammation-related miRNAs expression using TNF-*α* inhibitor followed by astrocyte stimulation with MRP8.

*Group E*. Astrocytes are cultured without serum DMEM medium containing TNF-*α* inhibitor lenalidomide ((2 *μ*M), Selleckchem, USA) for 6 hours and then cultured with DMEM medium with LPS for 24 hours to detect the effect of TNF-*α* inhibition on the tested brain-enriched and inflammation-related miRNAs expression using TNF-*α* inhibitor followed by astrocyte stimulation with LPS. 

### 2.4. RNA Isolation from Astrocytes

For RNA isolation, the astrocytes were washed with 0.01 mM PBS to clean the medium first. Then 1 mL of Trizol per 1 × 10^6^ astrocytes was added and collected in 1.5 mL Eppendorf tubes. After addition of 0.2 mL chloroform, the aqueous phase was isolated using phase-lock tubes (Eppendorf, Hamburg, Germany). RNA was precipitated with 0.5 mL isopropyl alcohol, washed twice with 75% ethanol, and dissolved in nuclease-free water. The concentration and purity of RNA were determined at 260/280 nm using a nanodrop spectrophotometer (Ocean Optics, Dunedin, FL, USA).

### 2.5. Expressions of the Tested Brain-Enriched and Inflammation-Related miRNAs in the Different Astrocytes Groups by Quantitative PCR (qPCR)

cDNA synthesis was performed from the RNA extracted from the different prepared astrocyte groups using the One Step PrimeScript miR cDNA Synthesis Kit (TAKARA, Dalian, China) which includes three mixes (2X miRNA Reaction Buffer Mix, miR PrimeScript RT Enzyme Mix, and 0.1% BSA). A 10 *μ*L reaction contained 5 *μ*L 2X miR reaction buffer mix, 1 *μ*L miR PrimeScript RT enzyme mix, and 1 *μ*L 0.1% BSA. RNA 100 pg, and DEPC-treated water up to 10 *μ*L. The tubes were incubated at 37°C for 60 min; the reaction was terminated at 85°C for 5 sec, and then the reaction was held at 4°C. qPCR was performed using the SYBRR Premix Ex Taq II (TAKARA, Dalian, China) kit in triplicate. The 10 *μ*L PCR contained the following: 5 *μ*L SYBRR Premix Ex Taq II, 0.4 *μ*L Uni-miR qPCR Primer, 2 *μ*L miR specific primer for the tested miRNA (GeneCopoeia, USA), 1 *μ*L cDNA, and 1.6 *μ*L DEPC-treated water. The PCRs were incubated at 50°C for 2 min (UDG incubation) and 95°C for 30 sec, followed by 40 cycles of 95°C for 5 sec and 60°C for 30 sec, followed by melting curve analysis from 65.0 to 95.0°C (increment 0.5°C, 0:05). The relative expression levels for brain-enriched miRNAs (124, 134, 9, 132, and 138) and inflammations-related miRNAs (146a, 21, 181a, 221, and 221) were calculated using the comparative CT method. The expression of the U6 small nuclear RNA gene was used as an internal control.

### 2.6. TNF-*α* Expression by Quantitative PCR (qPCR) in the Different Astrocyte Groups

cDNA was generated from the different astrocyte groups using PrimeScript RT Reagent Kit (TAKARA, Dalian, China) which includes four reagents: 5X PrimeScriptTM Buffer (for real time), PrimeScriptTM RT Enzyme Mix I, Oligo dT Primer, and Random 6 mers. A 10 *μ*L reaction contained 2 *μ*L 5X PrimeScriptTM Buffer, 0.5 *μ*L PrimeScriptTM RT Enzyme Mix I, 0.5 *μ*L Oligo dT Primer, and 0.5 *μ*L Random 6 mers. RNA 100 pg–1 ng and RNase-free dH_2_O water up to 10 *μ*L. The tubes were incubated at 37°C for 15 min; the reactions were terminated at 85°C for 5 sec, and then the reactions were held at 4°C. TNF-*α* expressions were analyzed using SYBR Premix Ex Taq II (TAKARA). qPCR was performed in triplicate. The 10 *μ*L PCR contained the following: 5 *μ*L SYBRR Premix ExTaq II, 1 *μ*L Specific Primer TNF-*α*-F (Sangon, China), 1 *μ*L Specific Primer TNF-*α*-R (Sangon, China), 1 *μ*L cDNA, and 2 *μ*L DEPC-treated water. The PCRs were incubated at 50°C for 2 min (UDG incubation) and 95°C for 30 sec, followed by 40 cycles of 95°C for 5 sec and 60°C for 30 sec, followed by melting curve analysis from 65.0 to 95.0°C (increment 0.5°C, 0:05). The relative expression levels for TNF-*α* were calculated using the comparative CT method. *β*-Actin was used as an internal control.

### 2.7. Statistical Analysis

All of the data are expressed as means ± standard deviation (SD). A Student's *t*-test was performed to determine significant differences between two groups. One-way analysis of variance (ANOVA) was utilized to determine significant differences among multiple groups. *P* < 0.05 was considered to be statistically significant.

## 3. Results

### 3.1. Relative Expressions of Brain-Enriched and Inflammation-Related miRNAs in Astrocytes after Stimulation with MRP8 or LPS

**Figure 1:**
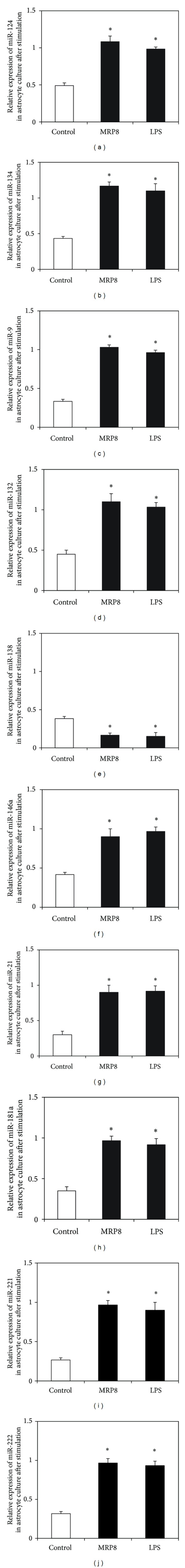
((a), (b), (c), (d), (f), (g), (h), (i), (j)) qPCR results showed significant upregulation of miRNAs (124, 134, 9, 132, 146a, 21, 181a, 221, and 222) in the astrocytes after stimulation with MRP8 and LPS, while miRNA-138 was significantly downregulated in the astrocytes after stimulation with MRP8 and LPS (e).

qPCR results showed significant upregulation of miRNAs (124, 134, 9, 132, 146a, 21, 181a, 221, and 222) in the astrocytes after stimulation with MRP8 or LPS compared to resting astrocytes, while miRNA-138 was significantly downregulated (means and SD values are given in Table 1). miRNAs expressions were normalized to that of the rat U6B small nuclear RNA gene (rnu6b) (Figures 1(a), 1(b), 1(c), 1(d), 1(e), 1(f), 1(g), 1(h), 1(i), and 1(j)).

### 3.2. TNF-*α* Relative Expression in the Astrocytes after Stimulation with MRP8 or LPS and Inhibition with Lenalidomide

**Figure 2:**
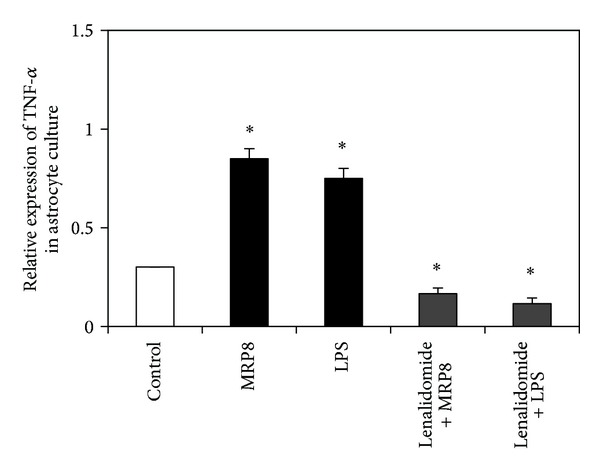
qPCR results showed significant upregulation of TNF-*α* in the astrocytes after stimulation with LPS and MRP8 and significant downregulation after inhibition with lenalidomide (TNF-*α* inhibitor) compared to stimulated astrocytes.

qPCR results showed significant upregulation of TNF-*α* in the astrocytes after stimulation with MRP8 or LPS compared to resting (resting mean 0.300 ± 0.0, MRP8 mean 0.850 ± 0.050, and LPS mean 0.750 ± 0.050,  *P* < 0.05). TNF-*α* expression showed significant downregulation after inhibition with TNF-*α* inhibitor (lenalidomide) compared to stimulated astrocytes (lenalidomide + MRP8 mean 0.166 ± 0.028; lenalidomide + LPS mean 0.116 ± 0.028,  *P* < 0.05). TNF-*α* expression was normalized to that of *β*-actin (Figure 2).

### 3.3. Relative Expressions of Brain-Enriched and Inflammation-Related miRNAs in the Astrocytes after TNF-*α* Inhibition with Lenalidomide Followed by Stimulation with MRP8 and LPS

**Figure 3:**
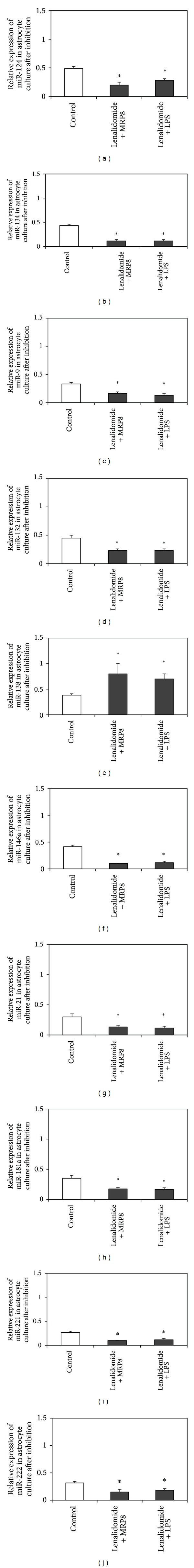
((a), (b), (c), (d), (f), (g), (h), (i), (j)) qPCR results showed significant downregulation of miRNAs (124, 134, 9, 132, 146a, 21, 181a, 221, and 222) after inhibition with lenalidomide compared to stimulated astrocytes, while miRNA-138 was significantly upregulated after inhibition with lenalidomide compared to stimulated astrocytes (e).

qPCR results showed significant downregulation of miRNAs (124, 134, 9, 132, 146a, 21, 181a, 221, and 222) in the astrocytes after TNF-*α* inhibition with lenalidomide compared to their expressions in the resting astrocytes while miRNA-138 showed the opposite pattern of expression (means and SD values are given in Table 1). MiRNAs expressions were normalized to that of the rat U6B small nuclear RNA gene (rnu6b) (Figures 3(a), 3(b), 3(c), 3(d), 3(e), 3(f), 3(g), 3(h), 3(i), and 3(j)).

## 4. Discussion

Astrocytes, constituting the major glial cell population in the CNS, are playing a crucial role in many developmental and physiological functions [19, 20]. Marked alterations in astrocyte function are a universal response to CNS disease or injury. Reactive astrocytes may provide neuroprotective effects in the early stage of the injury whereas at a later stage the formation of glial scar inhibits CNS regeneration [21]. 

The discovery of miRNAs functions reveals novel posttranscriptional regulation that controls or fine-tunes the transcriptional output. An increasing body of the literature supports the critical role of miRNAs in several biological processes of the CNS, as well as in the pathogenesis of different disorders including epilepsy, neurodegenerative, vascular, and neuroinflammatory disorders [22–26].

MiRNAs have critical roles in neuronal and astroglial development and cell fate decision. Deletion of miRNA-processing enzyme Dicer in neural progenitor cells was found to control the switch of neurogenesis to gliogenesis [27, 28]. Selective deletion of Dicer in the cerebellar astrocytes leads to global disruption of the astroglial miRNA biogenesis [29]. Moreover, miRNA-125b and miRNA-146a are involved in astroglial cell proliferation and in the innate immune and inflammatory response [30].

A better understanding of molecular mechanisms underlying astrocytes activation in response to stimulation with MRP8 and LPS, through detecting the changes in brain-enriched and inflammation-related miRNAs, may provide new step in understanding the pathogenesis of multiple brain disorders. In the first part of this experiment, we focused on the expressional changes in the brain-enriched miRNAs (124, 134, 9, 132, and 138) and inflammation-related miRNAs (146a, 21, 181a, 221, and 222) in the primary astrocyte culture in vitro, 24 hours after stimulation with MRP8 and LPS.

We found that brain-enriched miRNAs (124, 134, 9, and 132) showed significant upregulation in the astrocytes 24 hours after stimulation with MRP8 and LPS, while miRNA-138 takes the opposite pattern of expression. Mor et al. [13] have demonstrated a global shift in miRNA expression in astrocytes treated with LPS and IFN-*γ*. Although miRNA-124 is the most abundant brain-specific miRNA, there is a limited data about the importance of this miRNA in astrocyte function. Just very recently, Morel et al. [31] suggested that miRNA-124 can broadly modulate astroglial gene expression. MiRNA-124 has a very important role in maintaining the microglia in the quiescent state [32].

MiRNA-134 is brain-specific, implicated in the control of neuronal microstructure [33]. MiRNA-134 silencing exerts prolonged seizure suppressant and neuroprotective actions [34]. The exact function of miRNA-134 upregulation in the stimulated astrocytes needs further research.

Initially identified as brain-specific, miRNA-9 is a highly functional miRNA in brain development, which has been involved in the fine-tuning of nuclear factor Kappa-B- (NF-*κ*B-) dependent inflammatory response [35]. As an NF-*κ*B-mediated miRNA which plays a central role in CNS relevant stress and neuropathology, this may explain its upregulation in our experiment. 

MiRNA-132 was recently designated as a “NeurimmiR,” a class of miRs suggested to act as a crosstalk between the neuronal and immune system [36]. MiRNA-132 was upregulated in the activated astrocyte in our experiment; it was also upregulated in animal model of mesial temporal lobe epilepsy characterized by significant astrocyte activation [23]. 

In our experiment miRNA-138 was the only brain-enriched miRNA that was significantly downregulated in the stimulated astrocytes. MiRNA-138 was also downregulated in animal model after status epilepticus [37]. The exact role for this miRNA in astrocyte and the mechanism of its downregulation in response stimulation with MRP8 and LPS need further experiments.

Reactive astrocytes are playing essential roles in regulating CNS inflammation [21]. In response to different kinds of stimulation, reactive astrocytes can make many different kinds of molecules with either pro- or anti-inflammatory potential [21, 38]. Increasing evidence supports the involvement of miRNAs in the regulation of inflammation in human neurological disorders and various miRNAs are also considered to represent a new class of mediators of inflammation [11, 39].

We found that inflammation-related miRNAs (146a, 21, 181a, 221, and 222) showed significant upregulation in the astrocytes 24 hours after stimulation with MRP-8 and LPS. 

miRNA-146a was upregulated in the activated astrocyte in our work. Mor et al. [13] reported in their study activation of miRNA-146a in the astrocyte activation process in response to LPS and INF-*γ*. Upregulation of miRNA-146a has been also detected in active multiple sclerosis lesions [40], in human Alzheimer's disease brain [41] and in TLE [22, 42], suggesting a key role of this miRNA in governing astrocyte activation and function in these pathologies. Iyer et al. [43] found that miRNA-146a was induced as a negative-feedback regulator of the astrocyte-mediated inflammatory response and suggested a role for miRNA-146a-mediated regulation of inflammation in glial cells.

Due to the critical function of miRNA-21 in various inflammatory signaling pathways, miRNA-21 has become an attractive and promising target in inflammation-related diseases. We observed that the expression of miRNA-21 was upregulated in the stimulated astrocytes. Ziu et al. [14] found upregulation of miRNA-21 in the astrocytes exposed to OGD. Also miRNA-21 has a novel role in regulating astrocytic hypertrophy and glial scar progression after spinal cord injury [44]. 

MiRNA-181a which plays a critical role in the inflammatory response and development of immune system [45] was found to be also upregulated in the activated astrocytes. Reduction of miRNA-181a levels is associated with reduced cell death, reduced oxidative stress, and preserved mitochondrial function in astrocytes [46].

MiRNA-221 and miRNA-222 are two highly homologous miRNAs, whose dysregulations have been recently described in several types of human tumors. MiRNA-221 and miRNA-222 were upregulated in the activated astrocytes. MiRNA-221 was upregulated in the human astrocytic tumors [47]. Astrocytes activated with LPS and INF-*γ* showed upregulation of miR-222 [13].

TNF-*α* is a proinflammatory cytokine that not only acts upon astrocytes but is also produced by them in response to specific signals. Available data would suggest that TNF-*α* is involved in the induction of a reactive astrogliosis and may also play a role in CNS repair process. In our study we found significant upregulation of TNF-*α* expression when the astrocytes are stimulated with MRP8 or LPS while astrocytes treated with lenalidomide followed by stimulation with MRP8 or LPS showed significant downregulation of TNF-*α* expression.

In the second part of the present study, we examined for the first time the effect of lenalidomide (TNF-*α* inhibitor) on the expressions of the tested brain-enriched and inflammation-related miRNAs in the astrocytes.

We found that treating the astrocytes with lenalidomide for 6 hours, followed by stimulation with MRP8 or LPS for 24 hours, is associated with significant downregulation in miRNAs (124, 134, 9, 132, 146a, 21, 181a, 221, and 222) which are previously upregulated when the astrocytes were stimulated with MRP8 or LPS, while miRNA-138 surprisingly showed the opposite pattern of expression to be significantly upregulated in response to treatment with lenalidomide. This novel finding may indirectly link the changes that occur in the tested miRNAs in response to stimulation with MRP8 and LPS to the changes that occur in the TNF-*α* expression, which is reversed with treating the astrocytes with TNF-*α* inhibitor.

In conclusion, our study shows that exposure of astrocytes to MRP8 and LPS leads to significant changes in the expressions of brain-enriched and inflammation-related miRNAs and TNF-*α*. These changes are reversed when the astrocytes are treated with TNF-*α* inhibitor. These miRNAs may play a role in activation of the astrocytes and may be a novel target for cell-specific therapeutic interventions in multiple CNS diseases.

## Figures and Tables

**Table 1:** Means and SD of the brain-enriched and inflammation-related miRNAs in astrocytes after stimulation with (MRP8 and LPS) and inhibition of TNF-*α* with lenalidomide.

MiRNAs	Resting astrocytes	MRP8 stimulated	LPS stimulated	MRP8 + lenalidomide	LPS + lenalidomide
MiRNA-124	0.490 ± 0.036	1.083 ± 0.076	0.983 ± 0.028	0.200 ± 0.050	0.283 ± 0.028
MiRNA-134	0.433 ± 0.028	1.167 ± 0.057	1.100 ± 0.100	0.116 ± 0.028	0.116 ± 0.028
MiRNA-9	0.333 ± 0.028	1.033 ± 0.057	0.966 ± 0.057	0.166 ± 0.028	0.133 ± 0.028
MiRNA-132	0.450 ± 0.050	1.100 ± 0.100	1.033 ± 0.057	0.233 ± 0.028	0.233 ± 0.028
MiRNA-138	0.383 ± 0.028	0.166 ± 0.028	0.150 ± 0.050	0.800 ± 0.200	0.700 ± 0.100
MiRNA-146a	0.416 ± 0.028	0.900 ± 0.100	0.966 ± 0.057	0.100 ± 0.0	0.116 ± 0.028
MiRNA-21	0.300 ± 0.050	0.900 ± 0.100	0.916 ± 0.076	0.133 ± 0.028	0.116 ± 0.028
MiRNA-181a	0.350 ± 0.050	0.966 ± 0.057	0.916 ± 0.076	0.176 ± 0.025	0.166 ± 0.028
MiRNA-221	0.266 ± 0.028	0.966 ± 0.057	0.900 ± 0.100	0.100 ± 0.0	0.116 ± 0.028
MiRNA-222	0.316 ± 0.028	0.966 ± 0.057	0.933 ± 0.057	0.150 ± 0.050	0.183 ± 0.028
